# Modelling the effect of ephaptic coupling on spike propagation in peripheral nerve fibres

**DOI:** 10.1007/s00422-022-00934-9

**Published:** 2022-05-10

**Authors:** Helmut Schmidt, Thomas R. Knösche

**Affiliations:** 1 Max Planck Institute for Human Cognitive and Brain Sciences, Stephanstr. 1a, 04103 Leipzig, Germany; 2 Institute of Computer Science, The Czech Academy of Sciences, Pod Vodárenskou věží271/2, 182 07 Prague, Czech Republic; 3 Institute of Biomedical Engineering and Informatics, University of Technology Ilmenau, Gustav-Kirchhoff Str. 2, 98693 Ilmenau, Germany

**Keywords:** Peripheral nerves, Ephaptic coupling, Spike propagation, Synchronisation

## Abstract

Experimental and theoretical studies have shown that ephaptic coupling leads to the synchronisation and slowing down of spikes propagating along the axons within peripheral nerve bundles. However, the main focus thus far has been on a small number of identical axons, whereas realistic peripheral nerve bundles contain numerous axons with different diameters. Here, we present a computationally efficient spike propagation model, which captures the essential features of propagating spikes and their ephaptic interaction, and facilitates the theoretical investigation of spike volleys in large, heterogeneous fibre bundles. We first lay out the theoretical basis to describe how the spike in an active axon changes the membrane potential of a passive axon. These insights are then incorporated into the spike propagation model, which is calibrated with a biophysically realistic model based on Hodgkin–Huxley dynamics. The fully calibrated model is then applied to fibre bundles with a large number of axons and different types of axon diameter distributions. One key insight of this study is that the heterogeneity of the axonal diameters has a dispersive effect, and that a higher level of heterogeneity requires stronger ephaptic coupling to achieve full synchronisation between spikes.

## Introduction

Signal transmission in peripheral nerve fibres is based on the propagation of action potentials, or spikes, along the axonal membrane, which alters the electrophysiological properties of the extracellular medium and therefore influences nearby axons via so-called *ephaptic coupling* (Anastassiou et al. 2011; Buzsáki et al. 2012; Anastassiou and Koch 2015). Early experiments have demonstrated that in the presence of a highly resistive extracellular medium, action potentials travelling in two parallel, closely spaced axons synchronise and travel at a lower velocity than in the isolated case (Katz and Schmitt 1940; Rosenblueth 1941; Arvanitaki 1942; Marrazzi and Lorente de Nó 1944). This has been reproduced in theoretical work using numerical or analytical tools (Clark and Plonsey 1970; Bell 1981; Eilbeck et al. 1981; Barr and Plonsey 1992; Binczak et al. 2001; Bokil et al. 2001; Reutskiy et al. 2003; Maïna et al. 2015; Shneider and Pekker 2015; Goldwyn and Rinzel 2016; Schmidt and Knösche 2019; Sheheitli and Jirsa 2020; Capllonch-Juan and Sepulveda 2020). Both theoretical and experimental work, however, have been restricted thus far to a small number of identical axons due to the experimental or computational effort (with the exception of Capllonch-Juan and Sepulveda (2020)). In contrast, peripheral nerve bundles are composed of a relatively large number of nerve fibres, which follow a wide distribution of axonal diameters (Sanders 1947; Assaf et al. 2008; Ikeda and Oka 2012; Eichel et al. 2020). As there is an approximately linear relationship between the axon diameter and the velocity of a spike (Hursh 1939; Goldman and Albus 1968; Schmidt and Knösche 2019), one can expect that structural heterogeneity counteracts the synchronisation of spikes, akin to phase-coupled oscillators, where wider distributions of the angular frequencies require stronger coupling to achieve synchronisation (Kuramoto 1984; Pikovsky et al. 2001). The main difference between phase-coupled oscillators and ephaptically coupled spikes is that in the latter case the coupling is restricted to axonal segments close to the spikes, and that the lifetime of a spike is restricted to the time interval between spike generation and the spike reaching the distal end of the axon. Therefore, spike synchronisation depends crucially on the initial conditions, as demonstrated recently for white-matter fibre bundles (Schmidt et al. 2021). For this reason, we focus our analysis on initially synchronous spike volleys, in line with previous studies (Binczak et al. 2001; Reutskiy et al. 2003).

Our aim is to systematically study ephaptic coupling effects in peripheral nerves with large axon counts and distributed morphology. For this, we devise a simplified spike propagation model (SPM) that reduces the computational effort significantly as compared to solving models based on nonlinear partial differential equations. The SPM is inspired by phase-coupled oscillators in the sense that each spike is endowed with an intrinsic propagation velocity based on the morphology of the associated axon. Furthermore, each spike can be ascribed a coupling function that characterises the spatiotemporal profile of ephaptic coupling with spikes in nearby axons. The collective ephaptic coupling generated by all spikes in a fibre bundle then perturbs the intrinsic velocity of a single spike, which decelerates if the axonal membrane is hyperpolarised, and vice versa. In the resulting numerical scheme, the position of each spike is propagated based on its perturbed velocity. In turn, the positions and velocities of the spikes determine their mutual ephaptic coupling effects.

We begin our investigation by considering an exemplary bundle containing two axons, for which we develop the theoretical basis that can be extended to larger fibre bundles. First, we compute the effect that the spike in an active axon has on a nearby passive axon, which we do analytically under the assumption that the axons are homogeneous and that the perturbation of the passive axon can be captured by the linear terms of the cable equation. As most axons in peripheral nerves are myelinated, the assumption of homogeneity implies that the specific location of nodes of Ranvier is irrelevant. We calibrate the proposed SPM using a biophysically realistic model based on Hodgkin–Huxley dynamics.

The calibrated SPM is then used to study large axon bundles systematically, especially the interplay between axon diameter distribution and the strength of ephaptic coupling, with the latter having a synchronising effect, and the former a dispersive effect. The coupling strength is determined by the fibre density (i.e. relative volume occupied by nerve fibres), and the resistivity of the extracellular medium. The aim here is to identify conditions under which synchronisation can occur given different axon diameter distributions and structural parameters, especially fibre density.

## Ephaptic coupling between nerve fibres

In this section, we lay the theoretical basis of the SPM by considering fibre bundles with a small number of axons. First, we derive an analytical expression for the extracellular potential generated by a spike in an active axon. This is then used to compute the perturbation of the membrane potential of a passive axon (an axon that does not carry a spike) generated by a spike in a nearby active axon. The perturbation can be regarded as the ephaptic coupling between two nerve fibres. The resulting mathematical framework is then extended to the interaction between multiple axons. To reduce the computational load, we introduce a piecewise quadratic approximation of the spatial spike profile, which allows us to compute the ephaptic coupling function analytically. Lastly, we introduce the SPM and calibrate it by fitting free parameters to a biophysically realistic model based on the cable equation and Hodgkin–Huxley dynamics.

### Perturbation of the extracellular medium by a spike in a single axon

Axons may be regarded as core conductors (Rall 1977; Trayanova et al. 1990; Holt and Koch 1999), whereby the formation and propagation of spikes can be described by the one-dimensional cable equation:

1 $$\begin{aligned} C_m \frac{\partial V}{\partial t} = g_{\mathrm{ax}} \frac{\partial ^2}{\partial x^2} \phi _i - g_m V + I_{{\mathrm{HH}}}(V). \end{aligned}$$

The derivation of the cable equation is based on Kirchhoff’s first law, which states that the currents are balanced at any given location inside or outside the axon. Here, the membrane potential *V* is defined as the difference between intracellular and extracellular potential, $V = \phi _i - \phi _e$. If the extracellular medium is grounded, then $V = \phi _i$ and the traditional cable equation is recovered. In general, however, that assumption does not hold because of the finite size of the extracellular medium, and its finite conductivity (Tveito et al. 2017). The term on the left-hand side of Eq. (1) describes the capacitive currents across the axonal membrane and the myelin sheath, with $C_m$ being the capacitance of the membrane and myelin. The terms on the right-hand side of Eq. (1) describe resistive currents, of which the first term is the longitudinal intra-axonal resistance, which depends on the intracellular conductivity $\sigma _{\mathrm{ax}}$ and the cross-sectional area of the axon $A_{\mathrm{ax}} = \pi r_{\mathrm{ax}}^2$ via $g_{\mathrm{ax}} = \sigma _{\mathrm{ax}} A_{\mathrm{ax}}$. The second term is the passive resistive current across the membrane and myelin, with $g_m$ being the radial resistance of the myelin and the membrane, and the third term represents voltage-gated currents described by the Hodgkin–Huxley model.

Under the assumption that the boundary of the fibre bundle is perfectly insulating, the current balance outside an axon can be formulated in the equivalent manner:

2 $$\begin{aligned} C_m \frac{\partial V}{\partial t} = -g_{\mathrm{ex}} \frac{\partial ^2}{\partial x^2} \phi _e - g_m V + I_{{\mathrm{HH}}}(V). \end{aligned}$$

Here, the (longitudinal) extracellular resistance $g_{\mathrm{ex}}$ depends on the extracellular conductivity $\sigma _{\mathrm{ex}}$ and the cross-sectional area of the extracellular space, $A_{\mathrm{ex}}$. This relationship, however, is no longer applicable if multiple axons are in the same bundle. In this case, the extracellular potential depends on the capacitive and resistive currents generated by all axons, and we therefore find the following relationship for the current balance in the extracellular medium:

3 $$\begin{aligned} \sum _{n=1}^N C_m \frac{\partial V_n}{\partial t} = -g_{\mathrm{ex}} \frac{\partial ^2}{\partial x^2} \phi _e - \sum _{n=1}^N \left\{ g_m V_n - I_{{\mathrm{HH}}}(V_n) \right\} , \end{aligned}$$

with *N* being the total number of axons. It is easy to see from Eq. (3) that if the extracellular conductivity or the cross-sectional area of the extracellular medium increases (thus increasing $g_{\mathrm{ex}}$), then $\phi _e$ decreases (which has been demonstrated numerically in Tveito et al. (2017)).

Typically, spikes are measured as the spatiotemporal profile of the depolarisation of the membrane potential *V*. Here, we are interested in recovering a relationship between the extracellular potential $\phi _e$ and the membrane potential *V*. Combining Eq. (3) and Eq. (1), and using $\phi _{i,n} = V_n + \phi _e$, we obtain

4 $$\begin{aligned} \phi _e'' = -\frac{\sum _n g_{\mathrm{ax},n} V''_n}{g_{\mathrm{ex}}+\sum _n g_{\mathrm{ax},n}}. \end{aligned}$$

The terms $V_n ''$ and $\phi _e ''$ denote the second spatial derivative in the axial direction of $V_n$ and $\phi _e$. Although Eq. (4) is only an implicit representation of the extracellular potential via its second spatial derivative, we show that this expression is sufficient to compute the effect of a spike onto the membrane of a nearby axon. It is also obvious that the effect of axonal activity onto the extracellular medium is additive. In the following, we use the equivalent representation in terms of conductivities and fibre density. The fibre density $\rho$ can be defined as $\rho = g^{-2} A_{\mathrm{ax}} / (g^{-2} A_{\mathrm{ax}} + A_{\mathrm{ex}})$, where *g* is the g-ratio of the fibres, i.e. axonal diameter divided by fibre diameter (axon plus myelin). The perturbation of the extracellular medium by a spike in the $n^{{\mathrm{th}}}$ axon is thus given by

5 $$\begin{aligned} \phi _e'' = -\left( 1+\frac{\sigma _{\mathrm{ex}}}{\sigma _{\mathrm{ax}}} \frac{1-\rho }{g^2 \rho } \right) ^{-1} \frac{A_{\mathrm{ax},n} V_n''}{\sum _m A_{\mathrm{ax},m}}. \end{aligned}$$

We note here that we only consider axons with circular shape, therefore $A_{\mathrm{ax}}$ can be replaced by the square of the fibre diameter $d_n$.

### Perturbation of the membrane potential of a passive axon by a spike in a contiguous one

A spike can be regarded as a travelling wave along the axon. If the axon is sufficiently homogeneous, then this wave appears stationary in the co-moving frame. For simplicity, we assume here that the axons under consideration are either homogeneous, which would be the case for unmyelinated axons, or homogenised in the case of periodically myelinated axons. The term homogenised refers to the technique proposed by Basser (1993), which yields compound variables for the cable parameters that depend on the properties of myelinated and unmyelinated segments.

We transform the cable equation, Eq. (1), into the co-moving frame by setting $\xi = x - ct$, where *c* is the propagation velocity of the spike, which results in

6 $$\begin{aligned} -c \tau V' = \lambda ^2 (V'' + \phi _e'') - V + g_m^{-1} I_{{\mathrm{HH}}}(V). \end{aligned}$$

Here, we have made use again of $\phi _i = V + \phi _e$, as well as $\tau = C_m/g_m$ and $\lambda ^2 = g_{\mathrm{ax}}/g_m$, and $\cdot '$ indicates differentiation with respect to $\xi$. More specifically, $\tau$ is the homogenised time constant, and $\lambda$ is the homogenised length constant (Basser 1993):

7 $$\begin{aligned} \tau= & {} \lambda ^2 \left( \left( 1 - \frac{l}{L} \right) \frac{\tau _{\mathrm{myel}}}{\lambda _\mathrm{myel}^2} + \frac{l}{L} \frac{\tau _{\mathrm{node}}}{\lambda _{\mathrm{node}}^2} \right) , \end{aligned}$$

8 $$\begin{aligned} \lambda= & {} \left( \left( 1 - \frac{l}{L} \right) \lambda _\mathrm{myel}^{-2} + \frac{l}{L} \lambda _{\mathrm{node}}^{-2} \right) ^{-1/2}, \end{aligned}$$

where indices denote either myelinated segments with length *L*, or nodes of Ranvier with length *l*. The node and myelin-specific parameters were chosen to be $\tau _\mathrm{myel} = 0.47$ ms, $\tau _{\mathrm{node}} = 0.03$ ms, $\lambda _\mathrm{myel} = 1930 \sqrt{\text{ ln }(1/g)} d$, and $\lambda _{\mathrm{node}} = 55 \sqrt{d}$, with *d* being the axon diameter. These parameters are the same as in Schmidt and Knösche (2019), and all length scales are measured in $\mu$m. For simplicity, we set $l/L = 0.01$. Since the g-ratio is also held fixed at $g=0.6$, the only free parameter is the axon diameter *d*.

If the perturbation is sufficiently small, it will fail to elicit a spike in the passive axon, and the nonlinear term $I_{{\mathrm{HH}}}(V)$ can be neglected for simplicity. Thus we arrive at a linear ODE that describes the spatial profile of the perturbation in the passive axon:

9 $$\begin{aligned} - c \tau V_p' - \lambda ^2 V_p'' + V_p = \lambda ^2 \phi _e''. \end{aligned}$$

Here, $V_p$ indicates the perturbation of the membrane potential caused by $\phi _e''$. This is an inhomogeneous differential equation of second order. The homogeneous solution to (9) is found to be

10 $$\begin{aligned} V_{p,\mathrm{hom}} (\xi )= C_1 h_1(\xi ) + C_2 h_2(\xi ), \end{aligned}$$

where $h_1(\xi ) = \exp (\xi /\nu ^+)$ and $h_2(\xi ) = \exp (-\xi /\nu ^-)$ are the fundamental solutions to (9), with

11 $$\begin{aligned} \nu ^{\pm } = \frac{1}{2} \sqrt{ c^2 \tau ^2 + 4 \lambda ^2} \pm \frac{c \tau }{2}. \end{aligned}$$

Since the perturbations are localised, we require that $V_p \rightarrow 0$ as $\xi \rightarrow \pm \infty$, and therefore $V_{p,\mathrm{hom}} = 0$. Nevertheless, the fundamental solutions serve to identify the particular solution to (9), which is found by the method of variation of parameters. The particular solution can be posed as

12 $$\begin{aligned} V_{p,par} (\xi ) = \alpha _1(\xi ) h_1(\xi ) + \alpha _2(\xi ) h_2(\xi ), \end{aligned}$$

which is identical to the full solution to (9), $V_p(\xi )$. Since differentiation will yield only one equation for the two unknowns $\alpha _1(\xi )$ and $\alpha _2(\xi )$, we may make a further assumption regarding the solution structure:

13 $$\begin{aligned} \alpha _1'(\xi ) h_1(\xi ) + \alpha _2'(\xi ) h_2(\xi ) = 0. \end{aligned}$$

Differentiation of (12) and insertion of the resulting terms into (9) then yields

14 $$\begin{aligned} \alpha _1'(\xi ) h_1'(\xi ) + \alpha _2'(\xi ) h_2'(\xi ) = -\phi _e''(\xi ). \end{aligned}$$

Equations (13) and (14) pose a set of two linear equations that can be solved for $\alpha _1'(\xi )$ and $\alpha _2'(\xi )$. Subsequent integration results in

15 $$\begin{aligned} \alpha _1(\xi ) = -\int \frac{ \exp (-\xi /\nu ^+) \lambda ^2 \phi _e''(\xi )}{\sqrt{c^2 \tau ^2 + 4 \lambda ^2}} \mathrm{d} \xi , \end{aligned}$$

and

16 $$\begin{aligned} \alpha _2(\xi ) = \int \frac{ \exp (\xi /\nu ^-) \lambda ^2 \phi _e''(\xi )}{\sqrt{c^2 \tau ^2 + 4 \lambda ^2}} \mathrm{d} \xi . \end{aligned}$$

Since the integrands vanish at $\pm \infty$, we can pose these indefinite integrals as definite integrals on the interval $[\xi ,\infty )$ for $\alpha _1(\xi )$, and $(-\infty ,\xi ]$ for $\alpha _2(\xi )$ instead. This yields the following integral form for $V_p(\xi )$:

17 $$\begin{aligned} V_p(\xi )= & {} \int _{\xi }^{\infty } \frac{ \exp ((\xi -\zeta )/\nu ^+) \lambda ^2 \phi _e''(\zeta )}{\sqrt{c^2 \tau ^2 + 4 \lambda ^2}} \mathrm{d} \zeta \nonumber \\&+ \int _{-\infty }^{\xi } \frac{ \exp (-(\xi -\zeta )/\nu ^-) \lambda ^2 \phi _e''(\zeta )}{\sqrt{c^2 \tau ^2 + 4 \lambda ^2}} \mathrm{d} \zeta . \end{aligned}$$

An alternative representation is the following convolution integral:

18 $$\begin{aligned} V_p(x,t) = \int _{-\infty }^{\infty } w(x-y) \phi _{e}''(y,t) \text{ d }y, \end{aligned}$$

with the asymmetric convolution kernel given by

19 $$\begin{aligned} w(x) = \frac{\lambda ^2}{\sqrt{4 \lambda ^2 + c^2 \tau ^2}} \left\{ \begin{array}{ll} {\mathrm{e}}^{x/\nu ^+} &{} x \le 0, \\ {\mathrm{e}}^{-x/\nu ^-} &{} x > 0. \end{array} \right. \end{aligned}$$

**Fig. 1:**
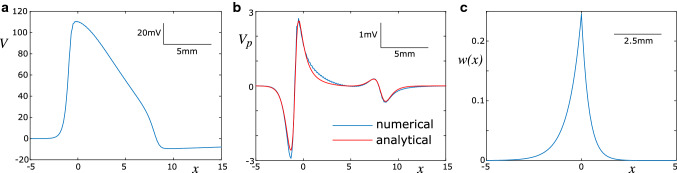
The perturbation in a passive axon can be described by a convolution of the curvature of the spike profile and an asymmetric kernel. **a** Spike profile at a particular point in time, computed numerically using the biophysical model (Sect. 2.5). The profile is representative for all stages of spike propagation. **b** Comparison of the perturbation in a passive axon obtained numerically with the biophysical model, and the convolution of the analytically derived integral kernel with the second derivative of the numerically obtained spike profile. **c** Profile of the asymmetric kernel. The kernel is compressed to the right (direction of propagation of the spike), and elongated to the left. Parameters: $\rho = 0.3$, $d = 1\,\mu$m, $c = 3.1$ m/s

The convolution kernel and integral are represented here in absolute space instead of the co-moving frame. In Fig. 1, we visualise the convolution kernel, and compare the perturbation of a passive axon that was obtained numerically, with the convolution of this kernel with the second derivative (curvature) of the spatial spike profile. The relatively small difference between the numerical and analytical result can be explained by nonlinear effects not taken into account in the convolution, and the homogenisation of the axonal parameters.

Thus far, we have considered a single spike that evokes a perturbation in a nearby passive axon [which may be regarded as a ‘test axon’ as in Goldwyn and Rinzel (2016)]. We now seek to extend the notation to accommodate the perturbations evoked by spikes in multiple axons travelling at different velocities. First, we consider the contribution to the perturbation of axon *i* by a spike in axon *j*:

20 $$\begin{aligned} V_p^{i,j}(x,t) = \int _{-\infty }^{\infty } w_{i,j}(x-y) \phi _{e,j}''(y,t) {\mathrm{d}}y, \end{aligned}$$

where $\phi _{e,j}$ is the contribution of the spike in axon *j* to the extracellular potential, and

21 $$\begin{aligned} w_{i,j}(x) = \frac{\lambda _i^2}{\sqrt{4 \lambda _i^2 + c_j^2 \tau _i^2}} \left\{ \begin{array}{ll} {\mathrm{e}}^{x/\nu ^+_{i,j}} &{} x \le 0, \\ {\mathrm{e}}^{-x/\nu ^-_{i,j}} &{} x > 0, \end{array} \right. \end{aligned}$$

is the convolution kernel specific to the interaction between axon *i* and axon *j*, with

22 $$\begin{aligned} \nu ^{\pm }_{i,j} = \frac{1}{2} \sqrt{ c_j^2 \tau _i^2 + 4 \lambda _i^2} \pm \frac{c_j \tau _i}{2}. \end{aligned}$$

Since we consider the linear regime (i.e. $I_{{\mathrm{HH}}}(V) = 0$), the perturbations are additive, and the full perturbation and extracellular potential are found to be

23 $$\begin{aligned} V_p^i = \sum _{j \in {\mathcal {A}}} V_p^{i,j}, \quad \phi _e = \sum _{j \in {\mathcal {A}}} \phi _{e,j}, \end{aligned}$$

where ${\mathcal {A}}$ is the set of all active, spike-carrying axons.

### Piecewise quadratic approximation of spike profiles

To reduce the computational load, we opt for an approximation of the spike profile that can be adjusted via a shape parameter $a_1$. In the time domain, the spike is parameterised as follows:

24 $$\begin{aligned} V(t) = \left\{ \begin{array}{ll} a_1 t^2 &{} \text{ if } \quad 0< t< t_{\max }/2\\ V_{\max } - a_1 (t-t_{\max })^2 &{} \text{ if } \quad t_{\max }/2< t< t_2\\ a_2 (t-1)^2 &{} \text{ if } \quad t_2< t < T_s\\ 0 &{} \text{ else }. \end{array} \right. \end{aligned}$$

The amplitude is set to $V_{\max } = 110$, and the other parameters can be related to $a_1$ by imposing smoothness conditions, which results in

25 $$\begin{aligned} t_{\max }= & {} \sqrt{2 V_{\max } / a_1}, \end{aligned}$$

26 $$\begin{aligned} t_2= & {} t_{\max } + \frac{V_{\max }}{a_1 (T_s-t_{\max })}, \end{aligned}$$

27 $$\begin{aligned} a_2= & {} \frac{V_{\max }}{(T_s-t_{\max })^2 - V_{\max } / a_1}. \end{aligned}$$

Thus, the spike has a total duration of $T_s$, and its shape can be varied between a triangular shape at large values of $a_1$, and a bell-shape at small values of $a_1$. The shape of the spike can then be translated into the spatial domain by calling $V(t) = V(\xi /c)$, which results from the co-moving frame transform.

**Fig. 2:**
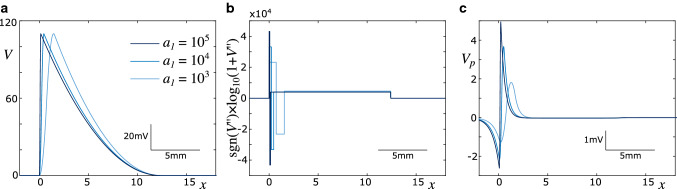
Piecewise quadratic approximation of spike profile. **a** The parameter $a_1$ controls the shape of the spike profile, where larger values correspond to faster depolarisation. **b** The curvature (second spatial derivative) is piecewise constant. It is depicted on a signed logarithmic scale for better comparison. The unit of the curvature is mV/mm$^2$. **c** Resulting perturbation in a passive axon. Parameters: $\rho = 0.3$, $d = 1\,\mu$m, $v = 3.1$ m/s, $T_s = 4$ ms

The piecewise quadratic approximation of the spike profile leads to a piecewise constant curvature, which allows us to compute the perturbation exerted by an active axon on its neighbouring axons analytically. The curvature is given by the second derivative:

28 $$\begin{aligned} V''(t) = \left\{ \begin{array}{ll} 2 a_1 &{} \text{ if } \quad 0< t< t_{\max }/2\\ - 2 a_1 &{} \text{ if } \quad t_{\max }/2< t< t_2\\ 2 a_2 &{} \text{ if } \quad t_2< t < T_s\\ 0 &{} \text{ else }. \end{array} \right. \end{aligned}$$

In the co-moving frame, this translates into

29 $$\begin{aligned} V''(\xi ) = \left\{ \begin{array}{ll} 2 a_1/c^2 &{}\quad \text{ if } \quad 0< \xi< c t_{\max }/2\\ - 2 a_1/c^2 &{}\quad \text{ if } \quad c t_{\max }/2< \xi< c t_2\\ 2 a_2/c^2 &{}\quad \text{ if } \quad c t_2< \xi < c T_s\\ 0 &{} \text{ else }. \end{array} \right. \end{aligned}$$

The quadratic approximation of the spike profile is shown in Fig. 2, alongside its second derivative and the resulting perturbation profile in a passive axon. The perturbation profile can be computed by inserting Eq. (29) into Eq. (5), and inserting the resulting expression for $\phi _e''$ into Eq. (20).

The complete mathematical expression for the perturbation in a passive axon driven by a spike in a second, active one is given by the following expression:

30 $$\begin{aligned} V_p^1(\xi ) = \left( 1+ \frac{\sigma _{\mathrm{ex}}}{\sigma _{\mathrm{ax}}} \frac{1-\rho }{g^2 \rho } \right) ^{-1} \frac{r_2^2}{r_1^2 + r_2^2} \frac{\lambda ^2_{1}}{\sqrt{4 \lambda ^2_{1} + c^2_2 \tau ^2}} G(\xi ),\nonumber \\ \end{aligned}$$

where $G(\xi )$ is a function describing the spatial profile of the perturbation:

31 $$\begin{aligned} G(\xi )= & {} -\frac{a_1}{c_2^2} F(\xi ;0,c_2 t_{\max }/2) + \frac{a_1}{c_2^2} F(\xi ;c_2 t_{\max }/2, c_2 t_2) \nonumber \\&- \frac{a_2}{c_2^2} F(\xi ;c_2 t_2, c_2 T_s), \end{aligned}$$

with

32 $$\begin{aligned}&F(\xi ;\xi _1,\xi _2) \nonumber \\&= \left\{ \begin{array}{ll} \nu ^+ \left( \mathrm{e}^{(\xi - \xi _1)/\nu ^+} - \mathrm{e}^{(\xi - \xi _2)/\nu ^+} \right) &{} \text{ if } \quad \xi \le \xi _1\\ \nu ^+ \left( 1 - \mathrm{e}^{(\xi - \xi _2)/\nu ^+} \right) + \nu ^- \left( 1 - \mathrm{e}^{-(\xi - \xi _1)/\nu ^-} \right) &{} \text{ if } \quad \xi _1<\xi <\xi _2\\ \nu ^- \left( \mathrm{e}^{-(\xi - \xi _2)/\nu ^-} - \mathrm{e}^{-(\xi - \xi _1)/\nu ^-} \right) &{} \text{ if } \quad \xi \ge \xi _2. \end{array} \right. \nonumber \\ \end{aligned}$$

These expressions can be readily translated into absolute space, which is omitted here for brevity.

### Calibration of spike propagation model

The spike propagation model was introduced previously for white-matter fibre bundles (Schmidt et al. 2021). Here, we adapt this model to accommodate the specific properties of peripheral nerve bundles. The major difference lies in how the extracellular potential is generated. While in white-matter fibre bundles one has to consider their radial extent due to their large diameter, peripheral bundles can be regarded as quasi-one-dimensional objects in terms of the parameterisation of the extracellular potential. For instance, if we consider a bundle containing 100 axons with diameters of approximately $1\,\upmu$m each, the resulting bundle diameter is approximately $10\,\upmu$m. The electrotonic length constant $\lambda$ of such axons, however, is approximately 1mm. This means that any effects not covered by the 1D representation of the axon bundle can be neglected, which is in line with the finding of Trayanova et al. (1990), whereby the 1D representation holds if the radius of the fibre bundle ($\approx$ 10$\,\upmu$m in our case, when $N=100$) is less than 0.03 times the length of the rising phase of an action potential (>1 mm in our case, cf. Fig. 1a). This implies that even at $N=1000$ this approximation would be valid. Furthermore, this holds if the diameters of the axons increase, since both the bundle diameter and the length of rising phase increase linearly with the axon diameter (the latter is due to the linear relationship between axon diameter and spike velocity in myelinated axons).

The core concept of the SPM is that a spike can be represented by its position on the axon, and its velocity. We remark here that the number of spikes in the SPM is preserved, i.e. that spikes are neither created (‘ectopic spikes’) nor annihilated by the ephaptic coupling. The velocity is considered constant along the axon in the unperturbed case. Perturbations of the membrane potential caused by spikes in other, contiguous axons, however, have an effect the propagation velocity, which is modelled in linearised form by:

33 $$\begin{aligned} v(x) = v_0 \left( 1 + \frac{1}{\gamma V_{\mathrm{thr}}} V_p(x) \right) . \end{aligned}$$

**Fig. 3:**
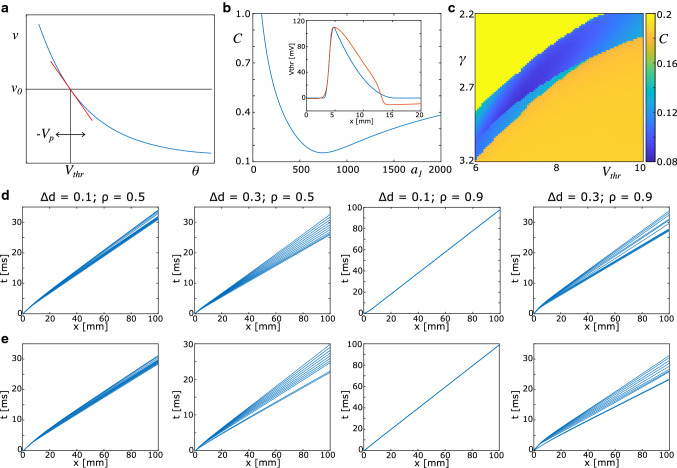
Calibration of spike propagation model. **a** An assumed nonlinear relationship between spike threshold and time lag between two reference points can be linearised around the default threshold value. The effect of perturbations of the membrane potential, and therefore of the spike threshold, on the time lag is then described in this linearised form. **b** The spike profile in the quadratic approximation is fitted to the spike profile of the biophysical model. The best fit occurs around $a_1 \approx 740$. **c** Cost function for varying values of $V_\mathrm{thr}$ and $\gamma$. The best fit is identified for the following set of parameters: $\gamma = 2.785$, $V_{\mathrm{thr}} = 7.05$. **d** Spike trajectories in the SPM. Spike volleys are initiated at $x=0$ and $t=0$. **e** Spike trajectories generated with the biophysical model

This is based on the assumption that the propagation velocity is determined by a putative spike threshold $\theta$ via $v = f(\theta )$, with $v_0 = f(V_\mathrm{thr})$ (Fig. 3a). Eq. (33) is then obtained by linearising $f(\theta )$ around $V_\mathrm{thr}$, where $\gamma = -f'(V_\mathrm{thr})$. The perturbation of the membrane potential, $V_p(x)$, is computed as shown in the previous section, which requires knowledge of the spike’s position. Finally, the position of the spike on the $i^{th}$ axon (each active axon carries one spike) is determined by

34 $$\begin{aligned} \dot{x}_i = v(x) \Big |_{x=x_i}. \end{aligned}$$

This means that the perturbation at the location of the spike determines the instantaneous propagation velocity. In general, Eq. (34) has to be solved numerically. For computational reasons, we consider the difference between spike positions to compute *v*(*x*):

35 $$\begin{aligned} v(x) \Big |_{x=x_i} = v_{0,i} \left( 1 + \frac{1}{\gamma V_\mathrm{thr}} \sum _j V_p^{i,j}(x_j-x_i+x_{{\mathrm{thr}}}) \right) .\nonumber \\ \end{aligned}$$

Since the spike position $x_i$ refers to the point where the membrane potential first deviates from zero, $x_{{\mathrm{thr}}}=\sqrt{V_{{\mathrm{thr}}} / a_1} v(x_i)$ is introduced to mark the position where the membrane potential first reaches $V_{{\mathrm{thr}}}$. Consequently, the parameters $\gamma$ and $V_{{\mathrm{thr}}}$ cannot be lumped together and have to be treated separately in the calibration.

In total, the SPM has three free parameters, which are the shape parameter $a_1$, and the spike threshold $V_\mathrm{thr}$ and the coupling parameter $\gamma$, which set how strongly the perturbation affects the propagation velocity. The spike threshold also defines the spike position along the spike profile, where the spike is sensitive to perturbations. (We note here that although there are two threshold crossings, we only consider the one along the rising phase, which determines the propagation velocity. The falling phase (‘tail’) of the spike is due to repolarisation processes, which do not affect the spike velocity.)

We first fit the shape parameter $a_1$ by minimising a cost function, which is defined as the sum of squares of the difference between the model spike profile and the spike profile generated with the biophysical model, see Fig. 3b. Only the rising phase of the spike profile is considered here, since the falling phase does not contribute significantly to the perturbation of the extracellular medium (cf. Fig. 1b). There is a clear minimum of the cost function at $a_1 = 740$, which yields a near perfect match of the spike profiles along the rising phase (Fig. 3b).

To identify realistic values for $\gamma$ and $V_\mathrm{thr}$, we first generate data for the propagation velocities in $N=10$ coupled axons using a biophysically realistic model, which is explained in detail in the next section. We use different parameter values of the fibre density and the fibre diameters. Specifically, the fibre density is chosen at $\rho \in \{ 0.5 \quad 0.6 \quad 0.7 \quad 0.8 \quad 0.9 \}$, and the fibre diameters are chosen to be $1\,\upmu$m for the smallest axon, and $(1+\Delta d)\upmu$m for the largest axon, with $\Delta d \in \{ 0.1 \quad 0.2 \quad 0.3 \}$. Thus, we fit the SPM to $N_p = 15$ parameter combinations $\mathbf {p}_n$ of $\rho$ and $\Delta d$.

To identify the best-fitting set of parameters, we define the following cost function that is to be minimised:

36 $$\begin{aligned} C(\mathbf {P}) = \sqrt{ \frac{1}{N_p N} \sum _{n=1}^{N_p} \sum _{i=1}^N \left( \frac{\tau _{\mathrm {SPM},i}(\mathbf {p}_n, \mathbf {P}) - \tau _{\mathrm {BPM},i}(\mathbf {p}_n, \mathbf {P})}{\tau _{\mathrm {BPM},i}(\mathbf {p}_n.\mathbf {P})}\right) ^2},\nonumber \\ \end{aligned}$$

where $\tau _{\mathrm {SPM},i}$ and $\tau _{\mathrm {BPM},i}$ are the axonal delays of the spike in the $i^{th}$ axon generated with the SPM and the biophysical model, respectively. This cost function characterises the model discrepancy for a given set of free parameters $\mathbf {P} = (\gamma , V_\mathrm{thr} )$.

In Fig. 3, we illustrate the cost function, which shows a clear minimum at $\gamma = 2.785$ and $V_\mathrm{thr} = 7.05$ mV. The discontinuities in the cost function are due to transitions from asynchrony to synchrony. This result indicates that the perturbation of the membrane potential, $V_p$, affects the propagation velocity quite strongly. We note here that in a previous work on white-matter fibre bundles (Schmidt et al. 2021), the optimal parameters were found to be $\gamma = 6$ and $V_\mathrm{thr} = 30$ mV. This discrepancy can be explained by the fact that there the extracellular potential $\phi _e$ was used to compute the perturbation, whereas here we use the perturbed membrane potential. The latter has a smaller amplitude due to axial currents, which is implicitly modelled by the convolution kernel.

To further illustrate the match between the SPM and the biophysical model, we plot spike trajectories generated with the SPM (Fig. 3d) for selected parameter combinations of $\rho$ and $\Delta d$, and compare them with the corresponding trajectories generated with the biophysical model (Fig. 3e).

### Biophysical model of spike propagation

The biophysical model is based on Eq. (1), as it provides a detailed description of the nonlinear voltage-gated currents. Specifically, we rewrite Eq. (1) as

37 $$\begin{aligned} \tau \frac{\partial V_n}{\partial t} = \lambda (V_n + \phi _e)'' - V + g_m^{-1} I_{{\mathrm{HH}}}(V_n). \end{aligned}$$

where $V_n$ is the membrane potential of the *n*th axon, and we insert Eq. (5) for $\phi _e''$. For numerical purposes, space is discretised into $100\,\upmu$m long segments. In this description, we distinguish between nodal and internodal segments. Internodal segments do not contain nodes of Ranvier, therefore we set $\lambda = \lambda _\mathrm{myel}$, $\tau = \tau _\mathrm{myel}$, and $I_{{\mathrm{HH}}}(V) = 0$. For nodal segments, we compute $\lambda$ and $\tau$ according to Eq. (8), except that we replace the internodal length *L* by the length of the segment.

Voltage-gated ion channels are only located at the nodal segments, and they obey the following equations:

38 $$\begin{aligned} I_{{\mathrm{HH}}}(V) = g_{{\mathrm{Na}}} m^3 h (e_{{\mathrm{Na}}} - V) + g_{{\mathrm{K}}} n^4 (e_{{\mathrm{K}}} - V), \nonumber \\ \end{aligned}$$

with the dynamics of the gating variables given by Hodgkin and Huxley (1952)

39 $$\begin{aligned} \dot{m}= & {} \frac{2.5-0.1 V}{\exp (2.5 - 0.1V) - 1} (1-m) - 4 \exp (-V/18) m, \nonumber \\ \end{aligned}$$

40 $$\begin{aligned} \dot{h}= & {} 0.07 \exp (-V/20) (1-h) - \frac{1}{\exp (3.0 -0.1 V) +1} h, \nonumber \\\end{aligned}$$

41 $$\begin{aligned} \dot{m}= & {} \frac{0.1 - 0.01 V}{\exp (1-0.1 V) -1} (1-n) - 0.125 \exp (-V/80) n.\nonumber \\ \end{aligned}$$

The reversal potentials in Eq. (38) are chosen to be $e_{{\mathrm{Na}}} = 115\,$mV, and $e_{{\mathrm{K}}} = -12\,$mV (Brill et al. 1977). While the maximum conductivities were set to $g_{{\mathrm{Na}}} = 1200$ ms/cm$^2$ and $g_{{\mathrm{K}}} = 90$ ms/cm$^2$ by Brill et al. (1977), we chose $g_{{\mathrm{Na}}} = 4800$ ms/cm$^2$ and $g_{{\mathrm{K}}} = 720$ ms/cm$^2$ to facilitate faster spike propagation. $g_m$ in Eq. (37) was set to 33 ms/cm$^2$, which corresponds to $\tau _{\mathrm{node}} = 0.03$ ms. The equations were solved using the forward Euler method with $\Delta t = 0.05\mu$s. To avoid boundary effects, the axons were padded with 200 nodal and internodal segments (20 mm in total) on either side. Neumann boundary conditions were applied to both ends, i.e. $V'(a) = V'(b) = 0$, with $a = -20$ mm and $b = 120$ mm.

## Ephaptic coupling in nerve bundles

In this section, we utilise the SPM to study the effect of fibre heterogeneity on the velocity of, and synchronisation between spikes. A special focus here is on fibre bundles with heterogeneous fibre diameters, which are distributed according to either a shifted, uniform distribution or a shifted alpha distribution. Of particular interest is the interplay between this heterogeneity and the strength of ephaptic coupling as expressed by the fibre density.

For simplicity, we focus here on the case of spike volleys that engage all axons in the fibre bundle, and that are completely synchronous initially (i.e. emission times are identical). The novelty here lies in the fact that the SPM reduces the computational effort as compared to the biophysical representation of the axon bundle, which allows us to model the interaction within spike volleys in large bundles. The numerical efficiency allows us to perform parameter screenings to describe the behaviour of the SPM in detail. Specifically, we vary the relative amount of extracellular space, which scales inversely with the amount of ephaptic coupling, and we vary the width of the distribution of axon diameters. The fibre bundles are 100 mm long and we record the axonal delays, which are measured as the time difference between spike initiation and spike termination. We compare two types of distributions: a uniform distribution, and a shifted alpha distribution.

### Uniform distribution of fibre diameters

The uniform distribution is set up as follows:

42 $$\begin{aligned} \rho (d) = \frac{1}{\Delta d} \Theta (d-1) \Theta (1+\Delta d -d), \end{aligned}$$

where $\Delta d$ is the width of the uniform distribution, and $\Theta (\cdot )$ is the Heaviside step function. The minimum diameter is fixed at $1\,\upmu$m, which results in a maximum diameter of $(1+\Delta d) \upmu$m in this distribution. The mean and standard deviation of this distribution is $1+\Delta d/2$ and $\Delta d/\sqrt{12}$, respectively. The coefficient of variation (standard deviation divided by mean) is thus $\Delta d/\sqrt{3}(2+\Delta d)$.

**Fig. 4:**
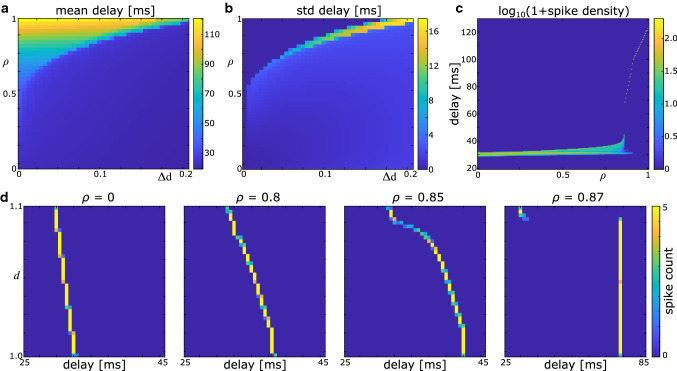
Delays in an axon bundle with uniform distribution of axonal diameters, as computed with the SPM. **a** Mean axonal delay for varying values of $\Delta d$ and $\rho$, $N=100$. **b** Standard deviation of delays, for same parameter range as in (**a**). **c** Logarithmic spike density showing the distribution of delays as $\rho$ increases. $\Delta d = 0.1$, $N = 200$. **d** 2D histograms of delays and axon diameters for varying values of $\rho$, in the absence of ephaptic coupling ($\rho = 0$) and around the transition to synchrony. $\Delta d = 0.1$, $N = 200$

In Fig. 4a, b, we show the effect of fibre density and diameter distribution on the mean and standard deviation of the axonal delays. At small values of $\Delta d$ and large values of $\rho$, spikes synchronise completely, as indicated by a standard deviation of delays close to zero. This is concurrent with an increase in the mean delay as the fibre density increases. The same synchronisation can be observed at larger values of $\Delta d$, yet a higher fibre density $\rho$ is required to achieve full synchronisation. Before full synchronisation sets in, the standard deviation of delays increases with $\rho$, which indicates that there is no (complete) synchronisation of the spikes within the volley. Rather, slow spikes form a synchronous cluster which slows down, while fast spikes remain asynchronous. We show a delay density plot in Fig. 4c, which illustrates the delay distribution across $\rho$ for $\Delta d = 0.1\,\upmu$m. Here, complete synchronisation occurs at $\rho \approx 0.86$, yet already at lower values one can observe clustering and slowing down of spikes. Figure 4d shows 2D histograms of delay distributions and axon diameters at the transition from asynchrony to synchrony. While there is a slight increase in delays with increasing $\rho$ in the asynchronous regime, there is a rapid increase in the delays when full synchronisation sets in.

### Fibre diameters from a shifted alpha distribution

To test whether these results depend on the type of diameter distribution, we perform the same analysis for a shifted alpha distribution:

43 $$\begin{aligned} \rho (d) = \alpha ^{-2} (d+1) \mathrm{e}^{-(d+1)/\alpha } \Theta (d-1). \end{aligned}$$

The minimum value in this distribution is $d=1$, but the maximum diameter depends on the number of axons *N*.

**Fig. 5:**
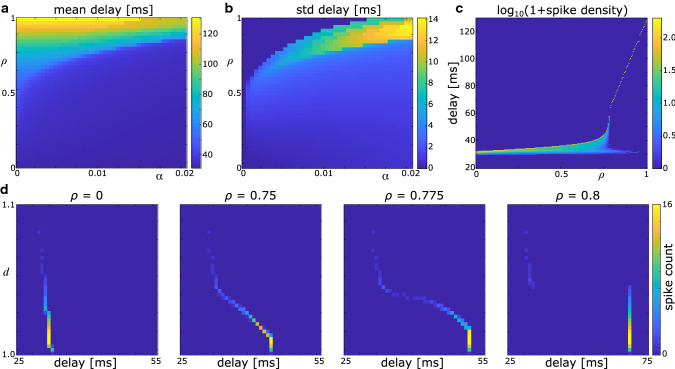
Delays in an axon bundle with axonal diameters obeying a shifted alpha distribution, as computed with the SPM. **a** Mean axonal delay for varying values of $\alpha$ and $\rho$, $N=100$. **b** Standard deviation of delays, for same parameter range as in (**a**). **c** Logarithmic spike density showing the distribution of delays as $\rho$ increases. $\alpha = 0.01$, $N = 200$. **d** 2D histograms of delays and axon diameters for varying values of $\rho$, in the absence of ephaptic coupling ($\rho = 0$) and around the transition to synchrony. $\alpha = 0.01$, $N = 200$

Once more, we find a parameter regime at small values of $\alpha$ and large values of $\rho$ where the spikes completely synchronise (Fig. 5a, b). At larger values of $\alpha$ we find again that synchronisation is only partial, in line with the results obtained for the uniform distribution of diameters. This indicates that the specific type of distribution is not important, rather the width of the distribution is essential for the types of synchronisation (complete or partial) between the spikes. To illustrate the route to synchronisation, we detail the results for $\alpha = 0.01$ in Fig. 5c. Once more, the spike density plot shows that across a wide range of $\rho$, synchronisation is partial, and only at around $\rho \approx 0.8$ it becomes complete. Again, a slow cluster forms at smaller values of $\rho$, which is seen in Fig. 5d.

### Comparison with biophysical model

We have identified parameters at which the spike volleys undergo complete synchronisation. While we have used the biophysical model to calibrate the SPM, that was done for a relatively small number of axons ($N=10$). We now focus on the transition to synchrony with uniformly distributed fibre diameters, and compare the delay distribution between the SPM and the biophysical model for $N=200$ axons. Due to the long simulation times for the biophysical model, we focus on three different values of $\rho$ at $\Delta d=0.1$.

**Fig. 6:**
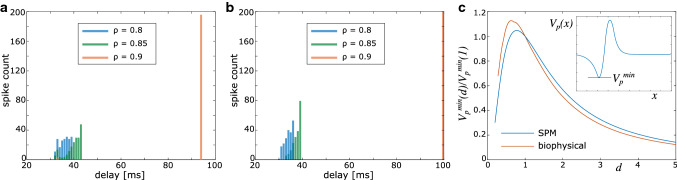
**a** Delay distribution in the SPM for uniformly distributed axon diameters, at different values of $\rho$ and $\Delta d = 0.1$, $N=200$. **b** Delay distribution generated with the biophysical model for the same parameters as in (**a**). **c** Estimate of relative coupling between axons of different diameters. A spike is generated in an axon with $d=1\,\upmu$m, and its effect on axons of varying diameters is computed by computing $V_p^{\min }$ (illustrated in inset) and plotting its amplitude relative to $V_p^{\min }$ at $d=1\,\upmu$m

The SPM predicts that at $\rho = 0.8$ and $\rho = 0.85$ the spikes do not fully synchronise, yet at $\rho = 0.85$ one can observe the formation of a slow, synchronised cluster. At $\rho = 0.9$, the spikes are fully synchronised (Fig. 6a). These results are confirmed by the biophysical model (Fig. 6b), albeit slow clusters in the asynchronous regime seem to be more strongly developed (as indicated by higher spike counts in the bin with highest delays).

It is interesting to note that slow spikes pertaining to small axons synchronise before the entire spike volley synchronises. To explain this effect, we investigate how strongly an axon is perturbed by a spike in a contiguous axon depending on its diameter. Since synchronous spikes experience the hyperpolarising phase of each other’s perturbation, we compute the minimum of $V_p$ for various diameters in response to a spike in an axon with $d=1\,\upmu$m. In Fig. 6c, we plot the amplitude of this minimum relative to the minimum in an axon with $d=1\,\upmu$m. Spikes are generated both with the SPM and the biophysical model, although $V_p$ is computed with the analytical approach (cf. Fig. 1b). Our results demonstrate unequivocally that the effect on smaller axons is stronger than on larger axons, which leads to earlier entrainment of slow spikes than of fast spikes.

## Discussion

The main contribution of the present study is to illustrate the effect of ephaptic coupling and fibre heterogeneity on the synchronisation of spike volleys in peripheral nerve bundles. The results confirm our initial hypothesis that while ephaptic coupling facilitates synchronisation, fibre heterogeneity counteracts this tendency. This is illustrated by the finding that for increasing fibre heterogeneity, the strength of ephaptic coupling (which increases with fibre density) necessary to produce full synchronisation within a spike volley also increases. This effect is independent of the specific type of fibre distribution chosen. At sufficiently high levels of heterogeneity, spike volleys no longer synchronise, even if the nerve bundle is completely filled with nerve fibre (fibre density $\rho = 1$). Nevertheless, in the absence of full synchronisation a more subtle phenomenon can be observed, which is the clustering and concurrent slowing down of spikes in small axons, while spikes in large axons do not synchronise. A possible explanation for this is that small axons experience a stronger perturbation of their membrane potential than large axons, cf. Fig. 6c. This clustering appears to be more prominent for the shifted alpha distribution, possibly due to the prominent peak at small axon diameters.

To obtain these results for large numbers of axons, we have adapted a spike propagation model (SPM) that was previously devised to simulate spike volleys in white-matter fibre bundles (Schmidt et al. 2021). The core assumption of the SPM is that the propagation of a spike is fully characterised by its intrinsic velocity (determined by structural and electrophysiological parameters of the corresponding nerve fibre), and the extracellular potential generated by spikes in nearby nerve fibres. The SPM therefore represents a much simpler model, without having to solve the nonlinear partial differential equation associated with the full biophysical model. Nonetheless, the SPM contains three free parameters that had to be calibrated using the biophysical model. We chose the scenario of ten ephaptically coupled nerve fibres to generate data for delays with the biophysical model, and the discrepancy between the data and the output of the SPM defined a cost function. Minimising this cost function, we were able to find an unambiguous optimal set of parameters for which the SPM matched best the biophysical results. Regardless of the specific choice of parameters, we surmise that synchronisation and clustering of spike volleys is a universal phenomenon in the SPM.

Some limitations apply to the SPM. One main limitation is that spike volleys travel across the fibre bundle without the possibility of generating (ectopic) spikes, or extinguishing them. In the present study, we only consider spike volleys that engage all axons, therefore only the latter would be a possibility. A mechanism of spike generation and extinction could be incorporated into the SPM by defining conditions involving the perturbation of the membrane potential. More precisely, if the membrane of a passive axon is sufficiently depolarised, then a spike is generated there; conversely, if the membrane is sufficiently hyperpolarised, a spike is extinguished. A previous study, based on a spatially extended FitzHugh–Nagumo model, has demonstrated the possibility of emerging ectopic spikes in ephaptically coupled axons (Sheheitli and Jirsa 2020).

Another limitation is that our study is based on a homogenised formulation of axonal morphology, which discounts the precise location and alignment of nodes of Ranvier. While a previous study has shown that the effect of ephaptic coupling was stronger when nodes are aligned, a staggered arrangement of nodes yielded the same qualitative results (Binczak et al. 2001). Node alignment could be taken into account in the SPM by generating data with the biophysical model for different levels of nodal alignment, and fitting the coupling parameter $\gamma$ to these values. Alternatively, theoretical considerations as in (Binczak et al. 2001) could serve to modulate the ephaptic coupling strength accordingly.

Our study considers relatively narrow axon diameter distributions to illustrate the effect of ephaptic coupling up to complete synchronisation. Peripheral nerves typically have a wide range of axon diameters, typically $1-10\,\upmu$m (Ikeda and Oka 2012; Eichel et al. 2020). In the context of our results, wide distributions prevent synchronisation and ensure exact temporal coding based on axon diameter, even if axons are densely packed. However, the clustering regime where only slow spikes synchronise could still be applicable, especially in fibre bundles with a selectivity for large or small axons, thus narrowing the distribution within the bundle. Here, the function of thicker and thinner axons could diverge: thinner axons might transmit convergent, synchronous information, whereas thicker axons might transmit time-critical information.

Finally, we would like to contemplate the possibility to confirm our results experimentally. While it is difficult to record spikes directly in peripheral nerves, it is easy to record the EP generated by nervous activity using surface electrodes. The EP of a spike volley can be computed using the line-source approximation (Holt and Koch 1999; McColgan et al. 2017). A typical EP waveform generated by a single spike is triphasic, with an initial hyperpolarisation, intermediate depolarisation, and final hyperpolarisation. In the case of spike volleys, this wave form is convolved by the spatial distribution of spikes in the volley. As a consequence, highly synchronised volleys will show a strong triphasic profile, whereas distributed volleys show weaker profiles. The clustering that we have observed would then likely result in a multiphasic EP profile, with a fast, low-amplitude response resulting from fast, asynchronous spikes, and a slow, high amplitude response resulting from slow, synchronised spikes. Interestingly, experiments show multiphasic EP profiles, which hints at the existence of such a clustering regime (Parker et al. 2018). Nevertheless, it is also possible that the multiphasic nature of these EPs is due to feedback mechanisms, and it requires more detailed modelling work to disentangle the different mechanisms.

## Declarations

### Code availability

The custom computer code that was created to generate the results presented in this manuscript is freely available under https://github.com/hschmidt82/ephaptic_peripheral_nerve.

## References

[CR1] Anastassiou C, Koch C (2015). Ephaptic coupling to endogenous electric field activity: why bother?. Curr Opin Neurobiol.

[CR2] Anastassiou C, Perin R, Markram H, Koch C (2011). Ephaptic coupling of cortical neurons. Nat Neurosci.

[CR3] Arvanitaki A (1942). Effects evoked in an axon by the activity of a contiguous one. J Neurophysiol.

[CR4] Assaf Y, Blumenfeld-Katzir T, Yovel Y, Basser P (2008). AxCaliber: a method for measuring axon diameter distribution from diffusion MRI. Magn Reson Med.

[CR5] Barr R, Plonsey R (1992). Electrophysiological interaction through the interstitial space between adjacent unmyelinated fibers. Biophys J.

[CR6] Basser P (1993). Cable equation for a myelinated axon derived from its microstructure. Med Biol Eng Comput.

[CR7] Bell J (1981). Modelling parallel, unmyelinated axons: pulse trapping and ephaptic transmission. SIAM J Appl Math.

[CR8] Binczak S, Eilbeck J, Scott A (2001). Ephaptic coupling of myelinated nerve fibers. Physica D.

[CR9] Bokil H, Laaris N, Blinder K, Ennis M, Keller A (2001). Ephaptic interactions in the mammalian olfactory system. J Neurosci.

[CR10] Brill M, Waxman S, Moore J, Joyner R (1977). Conduction velocity and spike configuration in myelinated fibres: computed dependence on internode distance. J Neurol Neurosurg Psychiatry.

[CR11] Buzsáki G, Anastassiou C, Koch C (2012). The origin of extracellular fields and currents—EEG, ECoG, LFP and spikes. Nat Rev Neurosci.

[CR12] Capllonch-Juan M, Sepulveda F (2020). Modelling the effects of ephaptic coupling on selectivity and response patterns during artificial stimulation of peripheral nerves. PLoS Comput Biol.

[CR13] Clark J, Plonsey R (1970). A mathematical study of nerve interaction. Biophys J.

[CR14] Eichel M, Gargareta VI, D’Este E, Fledrich R, Kungl T, Buscham T, Lüders K, Miracle C, Jung R, Distler U, Kusch K, Möbius W, Hülsmann S, Tenzer S, Nave KA, Werner H (2020). CMTM6 expressed on the Adaxonal Schwann cell surface restricts axonal diameters in peripheral nerves. Nat Commun.

[CR15] Eilbeck J, Luzader S, Scott A (1981). Pulse evolution on coupled nerve fibres. Bull Math Biol.

[CR16] Goldman L, Albus J (1968). Computation of impulse conduction in myelinated fibers; theoretical basis of the velocity-diameter relation. Biophys J.

[CR17] Goldwyn J, Rinzel J (2016). Neuronal coupling by endogenous electric fields: cable theory and applications to coincidence detector neurons in the auditory brain stem. J Neurophysiol.

[CR18] Hodgkin A, Huxley A (1952). A quantitative description of membrane current and its application to conduction and excitation in nerve. J Physiol.

[CR19] Holt G, Koch C (1999). Electrical interactions via the extracellular potential near cell bodies. J Comput Neurosci.

[CR20] Hursh J (1939). Conduction velocity and diameter of nerve fibers. Am J Physiol.

[CR21] Ikeda M, Oka Y (2012). The relationship between nerve conduction velocity and fiber morphology during peripheral nerve regeneration. Brain Behav.

[CR22] Katz B, Schmitt O (1940). Electric interaction between two adjacent nerve fibres. J Physiol.

[CR23] Kuramoto Y (1984). Chemical oscillations, waves, and turbulence.

[CR24] Maïna I, Tabi C, Fouda HE, Mohamadou A, Kofané T (2015) Discrete impulses in ephaptically coupled nerve fibers. Chaos 25:043118. 10.1063/1.4919077 10.1063/1.4919077 25933666

[CR25] Marrazzi A, Lorente de Nó R (1944). Interaction of neighbouring fibres in myelinated nerve. J Neurophysiol.

[CR26] McColgan T, Liu J, Kuokkanen P, Carr C, Wagner H, Kempter R (2017) Dipolar extracellular potentials generated by axonal projections. eLife 6:e26106. 10.7554/eLife.26106 10.7554/eLife.26106 PMC5617635 28871959

[CR27] Parker J, Shariati N, Karantonis D (2018). Electrically evoked compound action potential recording in peripheral nerves. Bioelectron Med.

[CR28] Pikovsky A, Rosenblum M, Kurths J (2001) Synchronization: a universal concept in nonlinear science. Cambridge University Press

[CR29] Rall W (1977) Core conductor theory and cable properties of neurons. In: Poeter R (ed) Handbook of physiology: the nervous system, vol 3. American Physiological Society, Bethesda, pp 39–97

[CR30] Reutskiy S, Rossoni E, Tirozzi B (2003). Conduction in bundles of demyelinated nerve fibers: computer simulation. Biol Cybern.

[CR31] Rosenblueth A (1941). The stimulation of myelinated axons by nerve impulses in adjacent myelinated axons. Am J Physiol.

[CR32] Sanders F (1947). The thickness of the myelin sheaths of normal and regenerating peripheral nerve fibres. Proc Roy Soc Lond Ser B.

[CR33] Schmidt H, Knösche T (2019). Action potential propagation and synchronisation in myelinated axons. PLoS Comput Biol.

[CR34] Schmidt H, Hahn G, Deco G, Knosche TR (2021). Ephaptic coupling in white matter fibre bundles modulates axonal transmission delays. PLOS Comput Biol.

[CR35] Sheheitli H, Jirsa V (2020). A mathematical model of ephaptic interactions in neuronal fiber pathways: could there be more than transmission along the tracts?. Network Neurosci.

[CR36] Shneider M, Pekker M (2015). Correlation of action potentials in adjacent neurons. Phys Biol.

[CR37] Trayanova N, Henriquez C, Plonsey R (1990). Limitations of approximate solutions for computing the extracellular potential of single fibers and bundle equivalents. IEEE Trans Biomed Eng.

[CR38] Tveit A, Jaeger K, Lines G, Paszkowski L, Edwards A, Maki-Marttunen T, Halnes G, Einevoll G (2017). An evaluation of the accuracy of classical models for computing the membrane potential and extracellular potential for neurons. Front Comput Neurosci.

